# Sirolimus-eluting stents: opposite *in vitro* effects on the clonogenic cell potential on a long-term exposure

**DOI:** 10.18632/oncotarget.27554

**Published:** 2020-08-04

**Authors:** Francesco Vasuri, Alessio Degiovanni, Mauro Gargiulo, William G. Thilly, Elena V. Gostjeva, Gianandrea Pasquinelli, Silvia Fittipaldi

**Affiliations:** ^1^Pathology Unit, Department of Experimental, Diagnostic and Specialty Medicine (DIMES), Bologna University, Bologna, Italy; ^2^Laboratory in Metakaryotic Biology (LIMB), Department of Biological Engineering, Massachusetts Institute of Technology, Cambridge, MA, USA; ^3^Vascular Surgery Unit, Department of Experimental, Diagnostic and Specialty Medicine (DIMES), Bologna University, Bologna, Italy

**Keywords:** atherosclerosis, cell proliferation, sirolimus, stents

## Abstract

We evaluated the long-term effects of sirolimus on three different cell *in vitro* models, cultured in physiological conditions mimicking sirolimus-eluted stent, in order to clarify the effectiveness of sirolimus in blocking cell proliferation and survival.

Three cells lines (WPMY-1 myofibroblasts, HT-29 colorectal adenocarcinoma, and U2OS osteosarcoma) were selected and growth in 10 ml of Minimum Essential Medium for 5 weeks with serial dilutions of sirolimus. The number of colonies and the number of cells per colony were counted.

As main result, the number of WPMY-1 surviving colonies increased in a dose-dependent manner when treated with sirolimus (*p* = 0.0011), while the number of U2OS colonies progressively decreased (*p* = 0.0011). The clonal capacity of HT-29 was not modified by the exposure to sirolimus (*p* = 0.6679).

In conclusion sirolimus showed the well-known cytostatic effect, but with an effect on clonogenic potential different among the different cell types. In the practice, the plaque typology and composition may influence the response to sirolimus and thus the effectiveness of eluted stent.

## INTRODUCTION

Rapamycin (sirolimus) is a widely used cytostatic drug blocking the cell cycle in the phase G1/S through the inhibition of the mammalian target of Rapamycin (mTOR) pathway, that has found several clinical applications, from immunosuppression in diabetes and organ transplantation to cancer therapy and drug-eluting stents (DES) [1–5]. Beside to its cytostatic activity, sirolimus was also discovered to protect normal human oral keratinocytes from apoptosis by activating autophagy, and to act as a basal stem cell keratinocyte-protecting drug in irradiated mice [6].

The effect of sirolimus on mesenchymal cells is unknown, but it is an important issue, since mesenchymal cells such as myofibroblasts and cells promoting vascular calcification play an important role in atherogenesis and vascular restenosis [7, 8]. In 2001 a preclinical study on a porcine model demonstrated that the use of a sirolimus-eluting stent (SES) reduced the in-stent neointimal hyperplasia of 35–50% [9]. As a consequence of these good results, the first clinical applications of SES in humans were performed in Sao Paulo and Rotterdam with good outcomes [10]. However, other clinical trials questioned the real effectiveness of SES, with contradictory evidences [11–15]. Sirolimus seems to block the proliferation and the migration of vascular smooth muscle cells [3], but we lack information concerning the effects on other cells composing atherosclerotic plaques.

The aim of the present paper is to evaluate the long-term effects of sirolimus, rather than short-term cell survival, on three different cell *in vitro* models, cultured in Minimum Essential Medium, which simulates physiological conditions (w/o CO2 and glucose [16, 17], in order to clarify the effectiveness of sirolimus in blocking cell proliferation and survival. Our hypothesis is that the efficacy of SES depends on the different cell composition within the atherosclerotic plaque.

## RESULTS

### Evaluation of sirolimus effects on clonal capacity and colony number (5 weeks)

**Figure 1:**
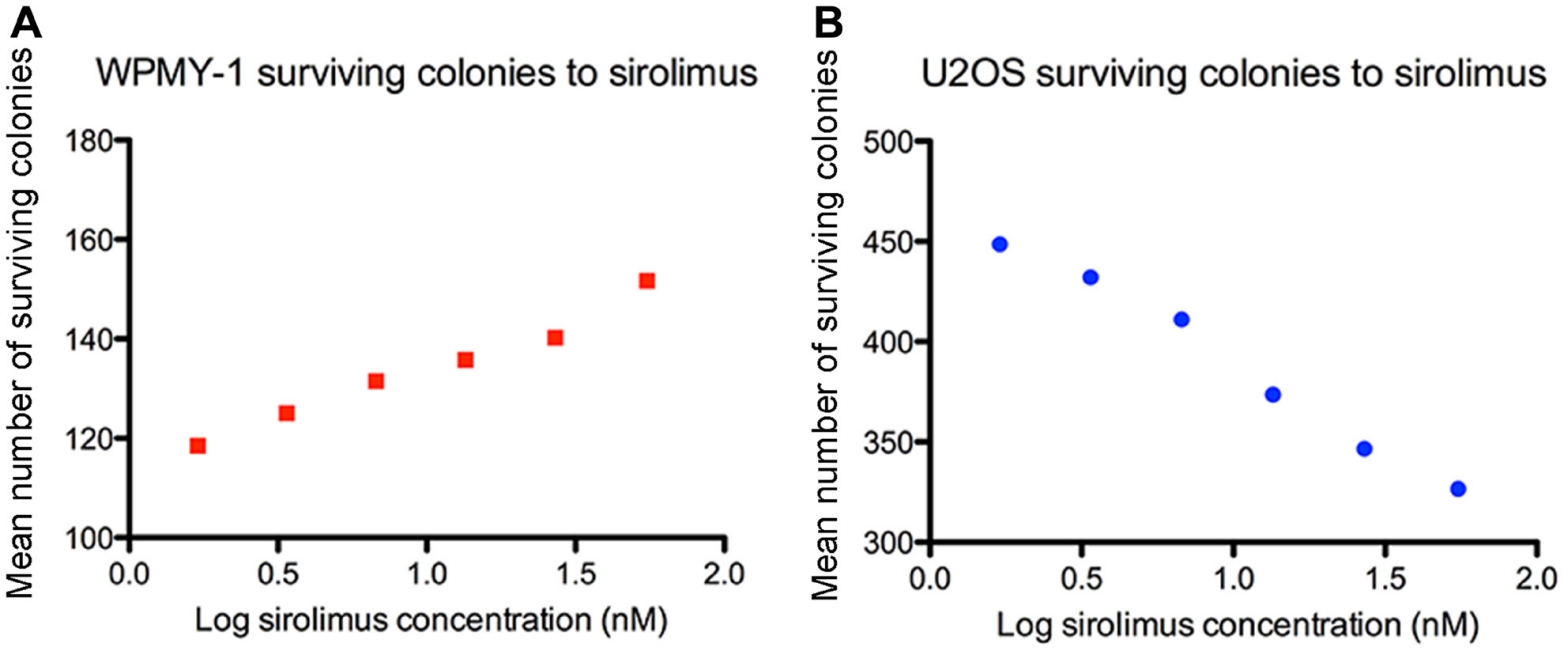
Concentration dose-response curve of sirolimus effect [55–1.7 nM] on the mean number of surviving colonies in WPMY-1 (**A**) and U2OS (**B**) cell lines after 2 weeks exposure and 3 weeks recovery.

The mean number of surviving WPMY-1 colonies after 2 weeks exposure to 55 nM sirolimus and 3 weeks of recovery was 151.7 ± 7.63 (range 145–160) colonies compared to 119.8 ± 3.86 (range114–122) colonies counted in untreated cells (Table 1), one way ANOVA *p* = 0.0011, Dunnett’s post-test *p* < 0.005). By analysing the effect of the exposure of WPMY-1 cells to sirolimus serial dilution (1.7 to 55.0 nmol, Figure 1A), it was observed that the number of WPMY-1 surviving colonies increased in a dose-dependent manner when treated with growing concentration of sirolimus (one way ANOVA *p* = 0.0011, post-test for linear trend: *p* < 0.0001).

**Table 1:** Mean number of surviving colonies treated with different concentration of sirolimus [55–1.7 nM] after 2 weeks exposure and 3 weeks recovery

Concentration sirolimus (nmol)	Mean number of colonies WPMY-1	Mean number of colonies HT-29	Mean number of colonies U2OS
55	151.7 ± 7.63	688.8 ± 47.65	326.5 ± 6.36
27	140.3 ± 1.71	609.0 ± 63.12	346.5 ± 4.95
12.5	135.8 ± 1.50	615.5 ± 16.13	373.5 ± 14.85
6.75	131.5 ± 4.65	646.3 ± 22.37	411.0 ± 24.04
3.38	125.0 ± 10.80	618.0 ± 18.81	432.0 ± 11.31
1.7	118.5 ± 2.38	605.3 ± 41.00	488.5 ± 12.02
0 (DMSO control)	119.8 ± 3.86	674.5 ± 50.58	412.5 ± 0.70

The mean number of surviving U20S colonies after 2 weeks exposure to 55 nM sirolimus and 3 weeks of recovery was 326.5 ± 6.36 (range 322–331) colonies compared to 412.5 ± 0.70 (range 412–413) colonies counted in untreated cells (Table 1, one-way ANOVA *p* = 0.0002, Dunnett’s post-test *p* < 0.005). By analysing the effect of the exposure of U2OS cells to sirolimus serial dilution (1.7 to 55.0 nmol, Figure 1B), it was observed that the number of U2OS surviving colonies decreased in a dose-dependent manner when treated with growing concentration of sirolimus (one way ANOVA *p* = 0.0011, post-test for linear trend: *p* < 0.0001).

The mean number of surviving HT-29 colonies after 2 weeks exposure to 55 nM sirolimus and 3 weeks of recovery was 688.8 ± 47.65 (range 630–739) colonies compared to 674.5 ± 50.58 (range 609–716) colonies counted in untreated cells (Table 1, one way ANOVA *p* = 0.0043, Dunnett’s post-test *p* < 0.05). By analysing the effect of the exposure of HT29 cells to sirolimus serial dilution (1.7 to 55.0 nmol), it was observed that the number of HT29 surviving colonies was not significantly correlated in a dose-dependent manner when treated with growing concentration of sirolimus (post-test for linear trend: *p* = 0.6679, *data not plotted*).

**Figure 2:**
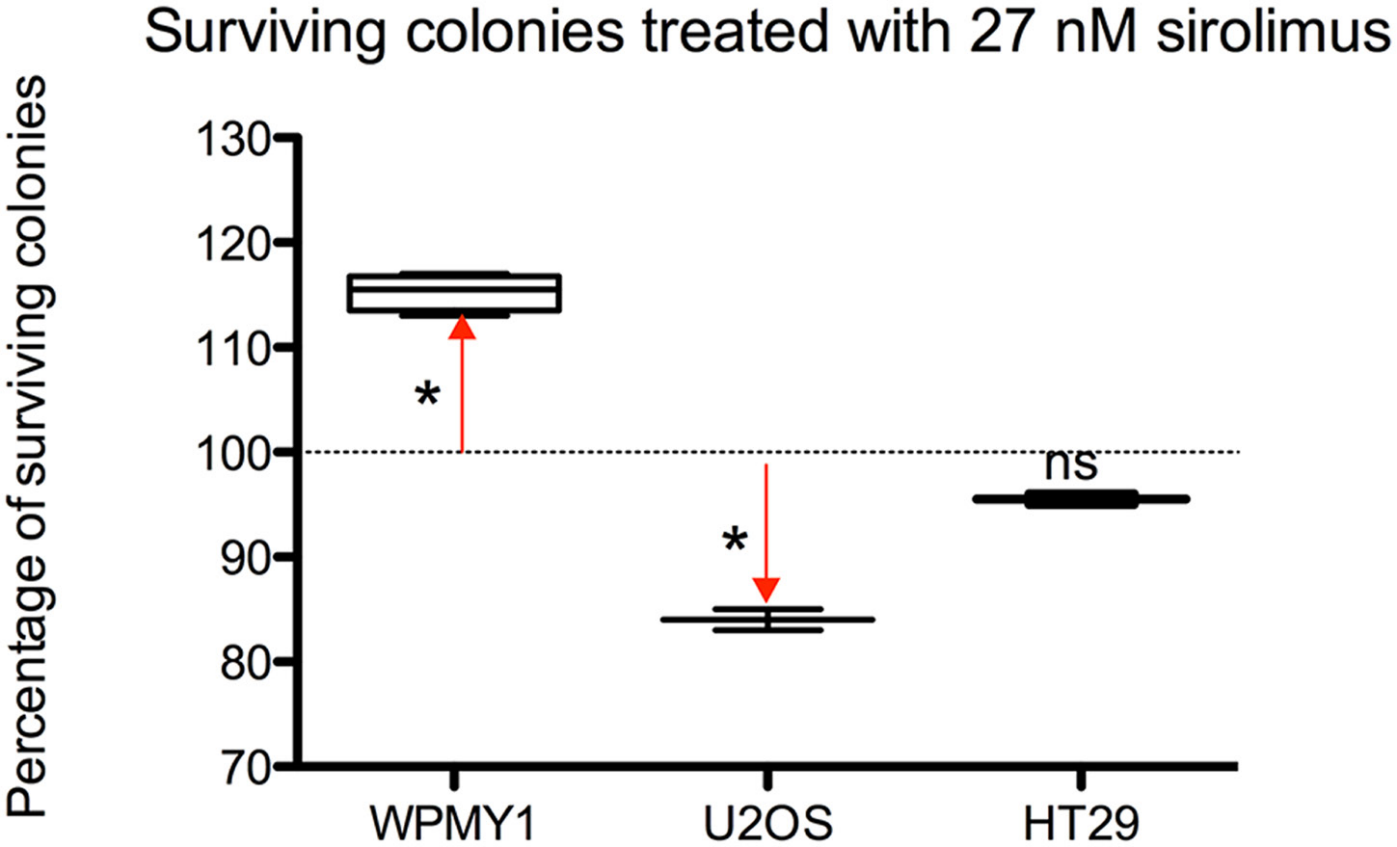
Differences among the 3 cell models of surviving colonies – expressed as a percentage-treated with 27 nM of sirolimus after 2 weeks exposure and 3 weeks recovery.

As shown in Figure 2, if we compare the effect of sirolimus at 27 nmol on the 3 cell lines, the percentage of surviving colonies –compared to untreated controls– after 2 weeks exposure and 3 weeks recovery was: 115.3% for WPMY-1, 84% for U2OS and 95.50% for HT29 (Dunnet’s post-test respectively *p* < 0.0005, *p* < 0.005 and *p* = ns).

### Confirmation of lack of sirolimus effects on clonal capacity - colony number- in HT-29 cells (8 days)

To clarify the results obtained at 5 weeks and to investigate whether the loss of variability in HT-29 cell lines was due to an extended culture time, HT-29 cells were treated with sirolimus for 8 days at a higher range of concentration (1 nM to 2560 nM). The mean number of surviving HT-29 colonies after 8 days exposure to 2560 nM sirolimus were 410.5 ± 48.79 (range 376–445) colonies compared to 485 ± 15.56 (range 475–497) colonies counted in untreated cells: the mean number of colonies at 2560 nM was not significantly different from untreated cells (Table 2, Dunnett’s post-test *P* value not significant). Indeed, the number of HT-29 surviving colonies after 8 days exposure was not significantly correlated in a dose-dependent manner when treated with serial dilution of sirolimus (post-test for linear trend: *p* = not significant).

**Table 2:** Mean number of surviving colonies treated with different concentration of sirolimus [2560–1.7 nM] after 8 days exposure

Concentration sirolimus (nmol)	Mean number of colonies HT-29
2560	410.5 ± 48.89
640	419.0 ± 5.65
320	427.5 ± 20.51
160	480.0 ± 46.67
80	549.5 ± 24.75
40	438.0 ± 41.01
20	399.5 ± 14.85
10	394.5 ± 27.58
1	380.0 ± 55.15
0 (DMSO control)	486.0 ± 15.86

HT-29 did not show a significant dose-dependent response to sirolimus in both short and long-term exposure. As we were interested in the clonal capacity of cells, we therefore excluded HT-29 from the next evaluation.

### Evaluation of the cytostatic effects of sirolimus (8 days)

**Figure 3:**
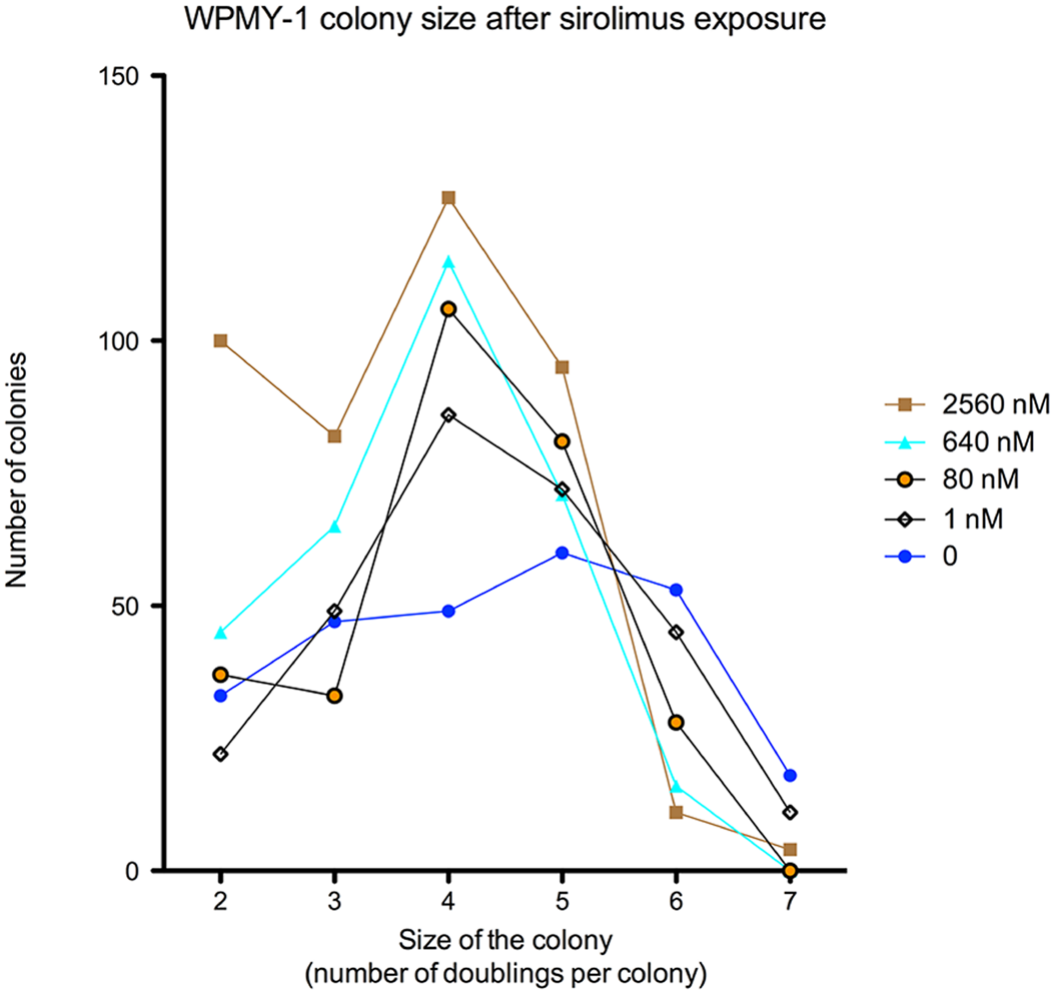
Concentration dose-response curves of sirolimus effect [2560–1 nM] on the number of cells per surviving colony in WPMY-1 cell line after 8 days exposure. To simplify graphical visualization only 5 concentration were represented.

To verify the cytostatic effects of sirolimus we evaluated the number of surviving cells in WPMY-1 and U2OS colonies treated with sirolimus (1 nM to 2560 nM). After 8 days exposure, the mean number of WPMY-1 cells per colonies ranged from 4 to 128 cells, corresponding to 2 to 7 doublings per cell plated after 8 days (Figure 3).

In the case of a WPMY-1 cell on monodisperse seeding, we expect 5 doublings after 8 days, equivalent to the formation of colonies composed of 32 cells (one doubling every 37 h).

In the untreated control, the majority of surviving cells encountered 5 doublings (number of colonies composed of 32 cells: 60 colonies (23.1%), two-way ANOVA, *p* < 0.001). After 8 days exposure to 2560 nM sirolimus, the majority of cells encountered only 4 doublings (number of colonies composed of 16 cells: 127 colonies (30.6%) in treated cells vs 49 colonies (18.8%) in untreated cell, (post-test Bonferroni, *p* < 0.01).

By analysing colonies composed of 64 cells, thus cells that encountered 6 doublings, we noticed that only 11 WPMY-1 colonies (2.6%) reached 6 doublings if treated with 2560 nM sirolimus, compared to 53 (20.0%) in untreated cells (*p* < 0.05).

As seen in Figure 3, the WPMY colony size (= number of cells) decreased in a dose-dependent manner when cells were treated with increased concentrations of sirolimus.

**Figure 4:**
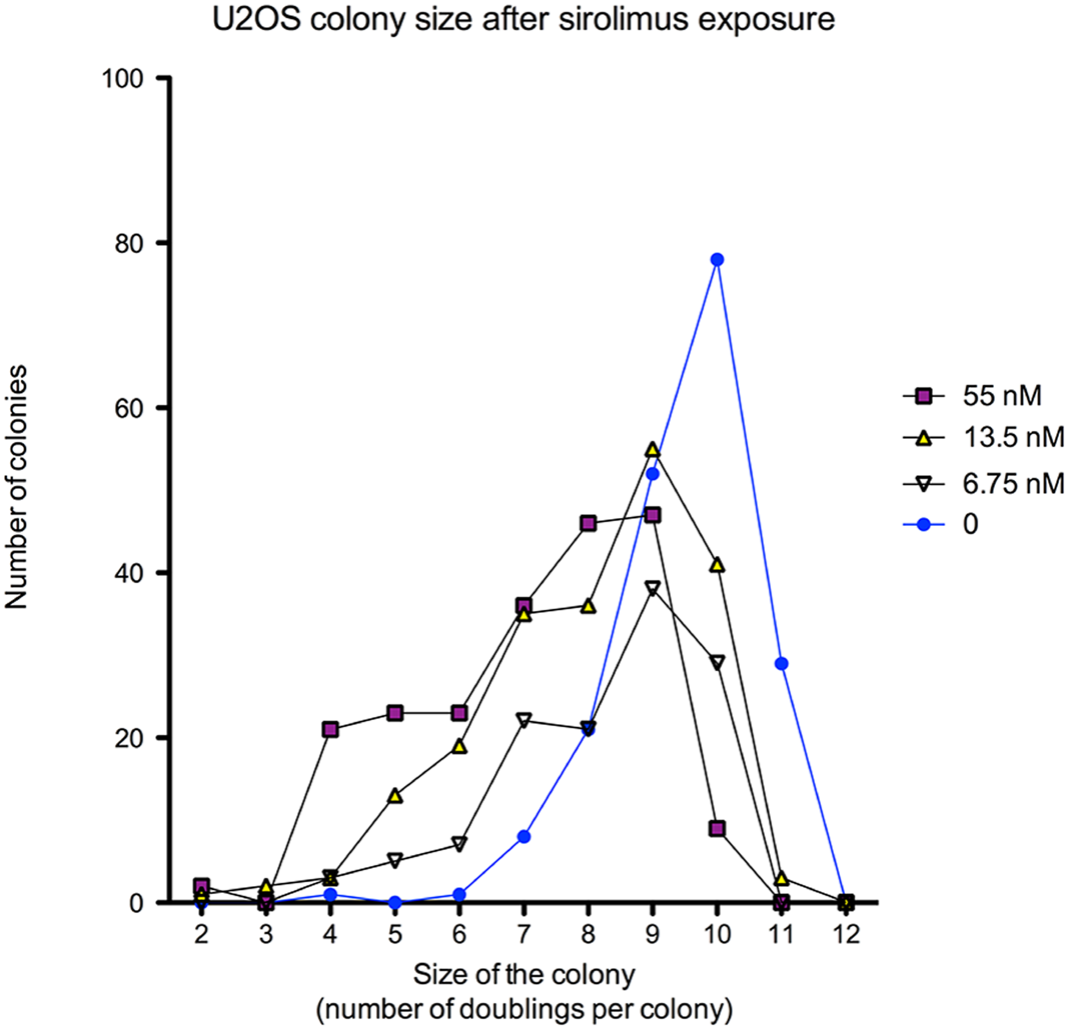
Concentration dose-response curves of sirolimus effect [55 nM–1 nM] on the number of cells per surviving colony in U2OS cell line after 2 weeks exposure.

To confirm the cytostatic effect of sirolimus also on U2OS cell lines, cells were counted directly on T75 flasks: the number of doublings for U2OS ranged from 2 to 11 (4 to 2048 cells per colony). The majority of untreated U2OS cells encountered 10 doublings (two-way ANOVA, *p* < 0.0001). The number of colonies composed of 1024 cells was 78 (41.05%) in untreated cell vs only 9 colonies (4.34%) at 55 nM sirolimus exposure (post-test Bonferroni, *p* < 0.01) (Figure 4).

As seen in Figure 4, the U2OS colony size (= number of cells) decreased in a dose-dependent manner when cells were treated with increased concentrations of sirolimus.

## DISCUSSION

Due to its anti-proliferative effect, sirolimus is a widely used drug to coat stent in order to reduce or prevent the in-stent neointimal hyperplasia [9]. However, according to some recent studies, it was seen that sirolimus could protect and extend the lifespan of cells and prolong the stem and progenitor cell population [22]. Contradictory data are also found in cancer: rapamycin seems to lead to cancer regression in some cases, and to a worse outcome in others. Many studies noted the tumours regrow when treatment stopped [23, 24].

The effect of sirolimus is still not clear on myofibroblasts, that are mesenchymal cells, important players in atherogenesis [7, 25]. The purpose of the present study was to clarify the effect of sirolimus on the clonal capacity of different cells in physiological condition and to investigate the effects on a long-term exposure. We therefore tested the short and long-term effects of sirolimus on cell division and clonal capacity by applying a new clonal assay approach and reproducing three different *in vitro* experimental models, two of which can mimic the cell types exposed to sirolimus in arteries.

We decided to use culture conditions without CO2 and glucose, in order to avoid the confounding effects on cell behaviour of these molecules, that could induce cell senescence and apoptosis [16, 17].

In the long-term exposure, it was observed that sirolimus has a dose-response effect in myofibroblasts (WPMY-1) and osteosarcoma cells (U2OS) but in the opposite way. The number of HT29 surviving colonies was not significantly correlated in a dose-dependent manner when treated with growing concentration of sirolimus. The clonal capacity of HT-29 was not modified by the exposure to sirolimus.

The finding that HT-29 cells are not affected by sirolimus was quite unexpected: other Authors originally described a cytostatic effect, even in hypoxic conditions [26]. However, in the previous work HT-29 proliferation was assayed after 24/48 hours sirolimus exposure, while in the present study we evaluate the effect of sirolimus at 8 days or 5 weeks. One can speculate that the short-term cytostatic effects observed early after sirolimus administration do not persist after 8 days. Moreover, HT-29 response to sirolimus is generally studied in combination with other drugs [27], while in the present study we evaluated the single-agent effect of sirolimus using doses that are consistent with the release *in vivo* from SES.

To assess the cytostatic effect of sirolimus, the number of cells composing each colony was counted: this gives an exact estimation of the effect of sirolimus in blocking cell proliferation. The cytostatic effect of sirolimus was confirmed on WPMY-1 and U2OS: the colony size (= number of cells) decreased in a dose-dependent manner when cells were treated with increased concentrations of sirolimus. The number of divisions of WPMY cells exposed to sirolimus was lower compared to cells free from sirolimus. The drug arrested temporarily the proliferation of WPMY-1 for 37 hours (1 doubling). This is in agreement with the well-known cytostatic effect of sirolimus [5].

Generally, sirolimus delays cell division 3 to 4 times depending on the dose exposure: at 40 nM equivalent to stent concentration, cell division is arrested for 2 cell cycles and then starts again. Clinically, it was confirmed that sirolimus can delay restenosis by arresting cell proliferation [10].

While the cytostatic effect is confirmed in WPMY-1 and U2OS, sirolimus treatment had a remarkable and completely unexpected effect on clonal capacity of myofibroblast: WPMY-1 treated with sirolimus had a higher percentage of surviving colonies. The protective effect on some cell lines was already observed by Iglesias-Bartolome that showed that sirolimus induced autophagy instead of apoptosis and delays senescence in epithelial stem cells, with no effect on proliferation of cancer cells [6]. One can speculate that SES delay restenosis by arresting cell proliferation, but once the drug is suspended, eventual arrested myofibroblasts present in an atherosclerotic vascular lesion might start to grow again with an even higher clonogenicity capacity.

Thus, the plaque typology and the different cell composition of the plaque, e. g., the presence of inflammatory cells, angiogenesis, prevalence of fibrosis, presence of osteogenic progenitors, may influence the response to sirolimus. Moreover, it is known that the clonal capacity varies between cells and we should consider this matter when evaluating the effectiveness of eluted stent [28]. Finally, additional mechanisms can have a role, such as amitotic cell division [29]. These mechanisms were also observed in human atherogenesis [30, 31] and could be fundamental to evaluate the *in vivo* effect of sirolimus too.

## MATERIALS AND METHODS

### Cell models and medium

To study sirolimus efficacy in blocking mitosis and stopping growth, we selected 3 cells lines: a model of myofibroblastic cell line, a cancer cell line and a human calcification model [18] (Table 3). Each cell line has a constant doubling time, and as a consequence a predictable cell growth:

WPMY-1: non tumorigenic, healthy myofibroblasts, doubling time 37 h.

HT-29: human colorectal adenocarcinoma, doubling time 24 h.

U2OS: human osteosarcoma, doubling time 48 h.

Cells were grown in 10 ml of Minimum Essential Medium (MEM, Gibco), a specific medium under the intellectual property of Prof. W. Thilly (LIMB, Dept. of Biological Engineering, MIT, Cambridge MA) and manufactured on-demand by Gibco [17]. This medium is free of D-glucose, antibiotics and sodium bicarbonate and contains D-fructose. For *in vitro* analysis we added L-glutamine 4 mM and 10% FBS.

**Table 3:** Characteristics of cell culture model

WPMY-1	HT-29	U2OS
Healthy myofibroblasts	Colon cancer	Osteosarcoma
Non tumorigenic	Tumorigenic	Tumorigenic
Doubling time 37 h (0.65 doubling/24 h)	Doubling time 24 h (1 doubling/24 h)	Doubling time 48 h (0.5 doubling/24 h)
→ Healthy model, key actors in fibrotic plaques formation	→ Cancer model	→ **Arterial active calcification model**

### Ranges of sirolimus concentrations

We used commercial sirolimus (Rapamycin, Sigma, 2.5 mg/ml = 2.74 nM, solubility in water RAP 2.6 ug/ml), with a range of serial dilution from 1.7 to 55.0 nmol. This choice depended on the following sources:

- The sirolimus concentrations in humans according to pharmacokinetics is 1.7 nmol [19].
- Effect of sirolimus on cell line at a range of 10 nM–50 nM [20].
- Calculation of sirolimus concentration on SES: before performing the experiments, the evaluation of the concentration of sirolimus delivered by SES was calculated as a function of the vascular cell area in contact to the surface of the coated stent [10, 21].

We know that the normal coating concentration of commercial SES is 140 ug sirolimus/cm^2^, for a total of 153 ug per surface of stent.

We assumed: 1 cell = 100 um^2^ and stent area = 1 cm^2^. Thus, one stent is in contact with 1 000 000 cells.

The maximum dose for 1000 cells corresponds to 153 ng in 3 ml, which are equivalent to 50 ng/ml (or 55 nmol, assuming that the MW rapamycin is 914.2 g/mol).

### Evaluation of sirolimus effects on clonal capacity and colony number at 5 weeks

After thawing, each cell line was synchronized for 3 weeks, seeded as a monodisperse (1000 cells/T75), treated with sirolimus for 5 weeks, fixed in Carnoy and stained with Cristal Violet 2%.

**Figure 5:**
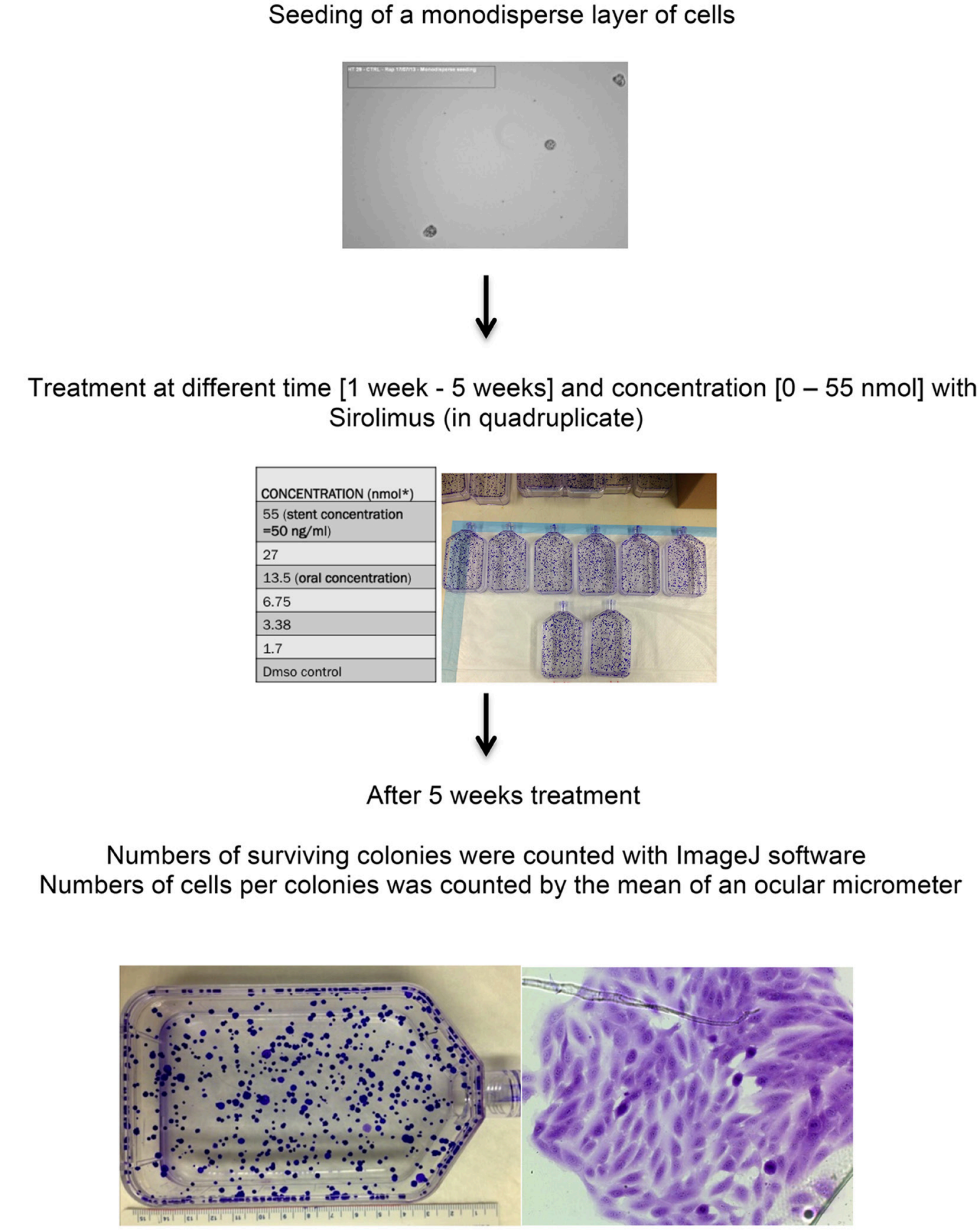
Experiment design for clonal assay in T75 flasks in cell lines treated with sirolimus at different time and concentration. Experiments parameters used were: - synchronized colonies, no use of CO2, fructose based media, - antibiotics free (streptomycin/penicillin) - long term exposition to sirolimus (5 weeks) - cells are grown for 1 months before any treatment - doubling time was constant for cell lines so growth of cells is predictable - monodisperse of cells.

Cells were seeded as a monodisperse (Figure 5A), treated with different ranges of sirolimus concentrations and let grown for a total of 5 weeks (Figure 5B). Sirolimus was removed after 2 weeks of exposure and cells grown for additional 3 weeks (Figure 5C). To be synchronized and before any treatment, cells were grown at least for one month until the doubling time (expressed as the number of doubling/24 h) was constant (WPMY-1: 0.65 doubling/24 h, HT29: 1 doubling/24 h, U20S: 0.5 doubling/24 h. Table 3).

A total of 84 T75 flasks were prepared for this work (Figure 5): for each of the 3 cell lines, 6 T75 were treated with 6 different sirolimus concentrations (55 nM, 27 nM, 13.5 nM, 6.75 nM, 3.38 nM, 1.7 nM) for 5 weeks, for a total of 21 T75. To verify if DMSO could affect cell viability, 2 different controls were prepared: one maximum concentration of DMSO used in treated cells and no DMSO.

Each experiment was conducted in quadruplicate.

We counted the number of surviving colonies and the number of cells composing each surviving colony exposed for 5 weeks at sirolimus 1.7–55.0 nM clinical range (Figure 5C). The number of surviving colonies indicates the clonal capacity, while the number of cells composing each colony is proportional to the number of cells doubling. This allows us to calculate the expected number of divisions for each cell line during exposition as a function of time. Also, this represents an exact estimation of the effect of sirolimus in blocking mitosis as well as the duration of the inhibition. Image J software was used for imaging quantification.

### Evaluation of sirolimus cytostatic properties at 8 days

To verify the cytostatic effects of sirolimus in WPMY-1, HT29 and U2OS we evaluated the number of surviving cells in each colony treated with different concentrations of sirolimus. Confluent cells at passage 85 (for WPMY-1), passage 50 (HT29) or passage 23 (U2OS) were seeded as a monodisperse on 6-well plates, each well containing a sterilized coverslip. For U2OS coverslip seeding for 8 days could not be performed, as U2OS are not suitable to grow on coverslip in these conditions: U2OS were counted directly on T75 flasks exposed to sirolimus. To verify if DMSO could affect cell viability, 4 controls were prepared, containing cells treated only with different dilutions (260, 1070 and 4280-fold) of DMSO, and one without DMSO. All experiments were carried out in duplicate.

After 8 days cells were fixed with Carnoy for 15 min and then with Ethanol 70’ for 1 h. Cells were therefore stained with Crystal Violet 2% (at rt for 1 h). Coverslips were washed with tap water for 10 min and mounted in PBS glycerol.

### Statistical analysis

Continuous variables were expressed as a means, standard deviation (SD) and ranges. Analyses of differences between more than two groups were performed with the one-way ANOVA test, followed by the comparison multiple post-test for linear trend. To compare mean colonies number for each concentration to control, Dunnett’s post-test was used. A *p* value < .05 was considered significant. To analyse and represent basic biostatistics, curve fitting, and scientific graphing of biological data Prism 5 software (GraphPad software, Inc) was used.

## Abbreviations

DES: drug-eluting stents
DMSO: dimethyl sulfoxide
mTOR: mammalian target of Rapamycin
SES: sirolimus-eluting stent
